# Use of alternative–complementary-medicine (CAM) in Calabrian children

**DOI:** 10.1186/1824-7288-38-70

**Published:** 2012-12-11

**Authors:** Teresa Rita Dolceamore, Federica Altomare, Francesco Zurlo, Roberto Miniero

**Affiliations:** 1 Primary Care Pediatrician, Crotone, Italy; 2 Università Magna Graecia di Catanzaro, Catanzaro, Italy

## Abstract

**Objective:**

The use of complementary and alternative medicine (CAM) has not been widely studied among children in Italy. ISTAT-2005 survey showed a prevalence of 10% concerning children treated with CAM. The lack of data about the use of CAM in pediatrics in the South of Italy aimed us to conduct an epidemiological inquiry in Calabria.

**Methods:**

The study has been conducted from 2009 and 2011 at the Pediatric Units of: University “Magna Graecia”- Catanzaro (CZ), Pugliese-Ciaccio Hospital-Catanzaro (CZ), Annunziata Hospital-Cosenza (CS), Jazzolino Hospital- ViboValentia (VV), Riuniti Hospitals- Reggio Calabria (RC) and San Giovanni di Dio Hospital- Crotone (KR). All information was collected through a questionnaire proposed to children’s parents admitted to these hospitals as out-patients or in-patients.

**Results:**

1387 parents were approached to complete the questionnaire. 21(1,5%) refused to answer. A total of 1366 questionnaire was analyzed: 378 at CZ , 450 at CS, 131 at KR, 201 at VV and 206 at RC, with a response rate of 98,5%. In total, the percentage of children using CAM varied from 18% in Crotone to 38% in Cosenza. The parents who used CAM for their children were older and with a higher education. Phytotherapy was preferred to homeopathy. The gastrointestinal pathologies and upper respiratory tract are those ones for which frequently parents recur to CAM. Of note we have not to disregard their use “ to strengthen” the immune system. In most of cases CAM have been prescribed by pediatrician.

**Conclusions:**

Our study remarks that the use of CAM is increased dramatically among the calabrian children in the last years as well as in other countries. Pediatricians need to improve their knowledge about CAM in order to better manage the parental attitude.

## Introduction

According to the National Centre for Complementary and Alternative Medicine (NCCAM), considered the leading institution providing studies and informations on these practices, Complementary and Alternative Medicine (CAM) are defined as “ preparations and practices that are not regarded as a part of conventional medicine and which may be used as complement or as an alternative to conventional medicine” [1].

In the USA the recourse of CAM is very extended in adults (up to 70% of them use some form of CAM) [2-5]. An increase of CAM (30% - 40% of the population) has been observed in Europe during the last two decades. The United Kingdom is the country which boasts the greatest historical tradition, but also in France and Germany the use of CAM is common among adults; in these countries CAM are regularly interconnected with Conventional Medicine, as they are integrated in the Universities and in the Public Health [2-5].

The European Project CAMbrella has been recently approved in order to build a research network for CAM; it encompass 16 academic research groups from 12 countries (Italy included) [6].

Available data for Italy are poor. Two researches of National Institute of Statistics (ISTAT), which were performed at the end of the 90’s and in 2005 recorded a prevalence of 15,5% and of 13,6%, respectively. Homeopathy was the most used kind of CAM, followed by hand treatments, phytotherapy and acupuncture. CAM are more common in the regions of Northern Italy [7]. In Tuscany, which represents the region where the unconventional treatments have a major spread and where they are recognized also by the National Health Service (SSN), the prevalence of CAM users is 15 to 20%, but 45% of the surveyed population consider useful at least one type of CAM [8,9].

International data about the use of CAM in pediatrics regard almost exclusively American and North European children. The prevalence of CAM use among children varies between 9% and 70% depending on the definition of CAM; the use is more frequent among those children with a parent who use CAM and among children suffering from chronic illness as tumors, juvenile rheumatoid arthritis, asthma and inflammatory bowel disease [10-28]. Far less is available concerning CAM use among children in the general population. A recent study in Germany remarked a rate of homeopathy use equal to 7,5% and phytotherapy one equal to 30,6% [29]. In Finnish population the prevalence of children CAM users was 10% and in Wales 41% [30,31].

Italian data about the use of CAM in pediatrics are few and they were achieved by researches in the Northern regions exclusively; this fact draws the line at the correct interpretation of the phenomenon [7-9,25,32]. The lack of data in the South of Italy aimed us to explore the patterns of CAM among a general population of Calabrian children, investigating the frequency and the type of CAM, the socio-demographic factors, the diseases treated with CAM and finally the perceived helpfulness and risk of side effect.

## Methods

The study has been carried out from 2009 to 2011 at the Pediatric Units of: University “Magna Graecia”-Catanzaro (CZ), Pugliese-Ciaccio Hospital- Catanzaro (CZ), Annunziata Hospital-Cosenza (CS), Jazzolino Hospital -ViboValentia (VV), Riuniti Hospitals- Reggio Calabria (RC) and San Giovanni di Dio Hospital- Crotone (KR).

All the information was collected through a questionnaire proposed to the parents of children admitted to these hospitals as out-patient or as in-patients. The definition of CAM is not homogeneous. In this survey only the CAM accepted by FNOMCEO were considered: homeopathy, phytotherapy, acupuncture, chiropractic, osteopathy, traditional Chinese medicine, antroposohycal medicine [33].

Data collection was based on a structured questionnaire, proposed *vis-à-vis* to the parents of young patients. It included questions about demographic characteristics of parents, diseases treated with of CAM, type of CAM, prescription, satisfaction and side effect observed.

The interviewers- students of 6th year University Medicine course, or young physician of the Pediatric School - have been trained in basic techniques of communication. The training of interviewers makes it more valid the comparison of data collected and their potential of information, as it is inevitable that the difference between different interviewers influences the responses to the questionnaire, through the effect known as "*expectation effect*": the training of interviewers was intended to minimize this effect on the research.

In the field of experimental sciences and statistics we refer to '*expectancy effect* (or *Rosenthal effect*) when referring to the distortion of the results of an experiment due to expectations that the researcher and / or experimental subjects have on the results of the experiment. [34]. With regard to the interviewer mentions the so called "interviewer error": a systematic error, related to the more or less aware selectivity of the data collection. Philipps and Clancy speak about *modeling effects* referring to the generality of the distortions produced by the interviewer using verbal and nonverbal (gestures, body posture, eye movements, tone of voice, pauses, etc.) [35]. The training of interviewers covered each of these aspects, with particular regard to the prosodic aspects, the use of the gaze, physical contact, managing questions, doubts and objections. The importance of interviewers training for the purposes of this research is also tied to the dual role of these. Responsibility of the interviewers was indeed also to give parents a detailed explanation of the topics examined in order to facilitate understanding and orientate the compilation (once again at the expense of comparability and therefore of their power and heuristic information). The training was conducted by a psychologist expert of communication. Before administration of the questionnaire a detailed description of the topic was provided to the parent. A written informed consent was obtained from the patient’s parents for publication of this report.

## Results

One thousand three-hundred eighty seven parents (primarily the mother) were approached to complete the questionnaire. Twenty one parents (1,5%) refused to answer, resulting in a total of 1366 questionnaires for analysis: 378 at CZ, 450 at CS, 131 at KR, 201 at VV and 206 at RC, with a cumulative response rate of 98,5%.

Outline the socio-demographic data of the parents interviewed. CAM users were more aged and with higher level of education than non-users. The difference was more evident for mother than for father. The features of the parents who filled in the questionnaire are summarized in Table 1.

**Table 1:** Characteristics of the study population

**Town**	**CZ**		**CS**		**VV**		**KR**		**RC**										
**CAM user** u	**Yes**	**No**	**Yes**	**No**	**Yes**	**No**	**Yes**	**No**	**Yes**	**No**
Mother’s mean age	40	35	39	35	31	36	36	36	36	36
Father’s mean age	48	40	42	41	41	38	41	39	39	39
**Mother’s instruction**										
Elementary/middle school	29%	46%	26%	41%	23%	47%	48%	50%	25%	48%
High school	53%	45%	50%	43%	65%	38%	39%	40%	36%	37%
University degree	18%	9%	23%	15%	12%	15%	13%	9%	39%	15%
**Father’s instruction**										
Elementary/middle school	39%	56%	35%	45%	49%	48%	39%	56%	26%	53%
High school	50%	37%	52%	44%	41%	36%	44%	34%	46%	37%
University degree	12%	7%	13%	11%	10%	14%	18%	8%	26%	7%
Patient’s mean age	6,0	2,0	7,4	7,4	5,2	5,5	7,3	6,7	5,2	6,4
Males%	50%	51%	58%	58%	57%	53%	43%	55%	46%	47%
Females %	50%	49%	42%	42%	43%	47%	56%	44%	54%	53%

**Figure 1:**
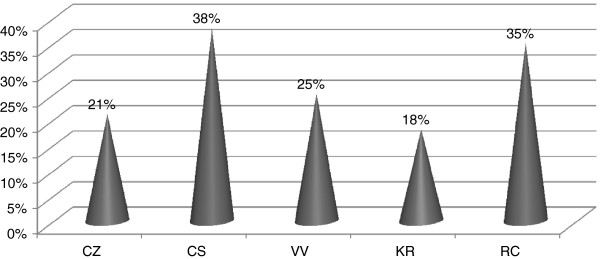
Percentage of patients treated with CAM.

**Figure 2:**
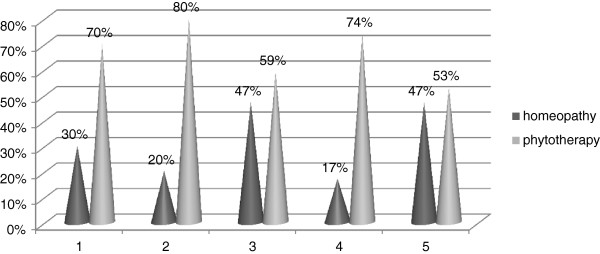
Type of CAM used.

In total, the percentage of children using CAM ranged from 18% in Crotone to 38% in Cosenza (Figure 1). Phytotherapy was more utilized than homeopathy. No other modalities of CAM were used (Figure 2). 

The most common illness treated with CAM were gastrointestinal diseases, upper respiratory tract diseases and dermatological disease; many parents used CAM “to strengthen” the immune system. Few patients were treated with CAM for chronic diseases (Table 2). 

**Table 2:** Diseases treated with CAM

**Town**	**CZ**	**CS**	**VV**	**KR**	**RC**
**Disease treated**					
Upper- airway diseases	58%	76%	41%	74%	46%
Gastrointestinal diseases	17%	41%	29%	13%	29%
To strenghthen immune sistem	13%	53%	30%	13%	32%
Teeth eruption pain	5%	3%	4%	-	3%
Dermatological diseases	13%	23%	12%	-	8%
Allergic disorders	-	3%	-	-	4%
Sleep disorders	-	-	8%	17%	1%
Minor injures	5%	8%	-	9%	4%
Other diseases	-	-	2%	13%	7%

The majority of parents feel that CAM must be prescribed by pediatrician; few of them self-initiated the use of CAM. The results are summarized in Table 3. 

**Table 3:** Medical prescription required by parents for CAM

**Town**	**CZ**	**CS**	**VV**	**KR**	**RC**
**Medical prescription**					
Always required	50%	64%	82%	48%	81%
Required according to the disease	47%	27%	16%	48%	21%
Unecessary	3%	9%	2%	4%	1%

All the CAM users, with the exception of 3, 5% of them in Cosenza who used only CAM for their children, were conventional medicine users too.

Perception of the efficacy of CAM was high in all interviewed. Referred side effects were few (Table 4) .

**Table 4:** Evaluation of therapeutic success with CAM

**Town**	**CZ**	**CS**	**VV**	**KR**	**RC**
In most of cases	65%	67%	51%	26%	57%
Some times	19%	28%	41%	61%	35%
Never	15%	5%	6%	13%	7%
Side effects	5%	2%	4%	0%	3%

## Discussion

In this multicenter survey we investigated the use of CAM in children through a questionnaire filled by parents of children admitted as in-patients or as out-patients in five General Hospitals in Calabria. The properties of the questionnaires to assess CAM use in pediatrics are still an open question and this may represent an important bias for interpreting the data of the literature. A recent review by April showed as none of CAM questionnaires have been thoroughly validated. This may be explained by the relative novelty of studies in this field. In fact is difficult to collect data in a standardized way since not only there is no agreement on the definition of CAM by researchers, but also for the fact that the parent’s perception of the product’s nature used may be confounding [36]. For these reasons, in order to reduce the bias toward the type of CAM, we decided to limit the number of CAM considered in the survey to the CAM reported by FNOMCEO [33]. Furthermore, most studies reported in literature used a self-administered questionnaire which has significantly limitation, first of all misinterpretation of terms and concepts. In order to minimize this second bias we preferred to administrate the questionnaire *vis-a-vis* properly training the interviewers. The percentage of non-compliant parents was very small (1,5%) and all the questionnaires filled in were evaluable. This confirms that the methodology we use (*vis-à-vis* questionnaire and trained interviewers) may represent a helpful model for these type of survey.

To the best of our knowledge, this is the first report concerning a large cohort of patients in the South of Italy. Our study shows that the use of CAM increased dramatically among the children living in southern Italy too and the percentage of patients treated with CAM is quite reaching the observed percentage in other European countries. In fact our study clearly shows that use of CAM in our Region is more common than expected, considering the results of ISTAT survey performed in 2005, which report a nationwide percentage of 13,6% of the population [7]. The explanation for the growing use of CAM in Calabria may involve the same factors of north Italian regions and other countries: most users are dissatisfied with conventional medicine; reject technological medicine; believe that CAM had no potential adverse effects; feel they need an holistic or natural approach rather than an approach focused on specific pathogenic process (generally emphasized by conventional medicine); search for a new consideration of those aspects often neglected – *inter alia* sense, communication and relation.

However in the light of the results coming from this inquiry it is possible to speculate that a slight difference exists among the five examined towns (from 18% of Crotone to 38% in Cosenza of CAM user). It is difficult to explain these differences, as socio-economics and socio-cultural back-ground are quite similar in the five populations, but it is interesting the fact that the percentage observed in Cosenza is quite similar to that one observed in our previous study in Turin (43%) [37].

Our study agrees with the current literature: the oldest and the most educated parents are those who trust to CAM [7-9,30,38]. These results might be interpreted in the light of a longer and personal experience in the use of CAM by the oldest parents and in the light of a wider economic checking among the most educated parents. A more qualified level of education corresponds to an economically best-paid profession and this is to influence the therapeutic choices made by parents. The unconventional therapies are considered more expensive and they aren’t refunded by the National Health System, with rare exceptions [8,9].

The ISTAT research, in 2005, reported the use of CAM more frequent in the oldest children [7]. Our research agrees with these data as it shows that especially the oldest children use them.

The majority of the parents exploit the unconventional therapies in the same way of the conventional medicine without laying upon it, with the exception of Cosenza, where an exiguous minority (3, 5%) of parents affirms they exclusively use CAM. So this common behavior agrees with the latest indications which aim at considering CAM as real complementary therapies instead of an alternative treatment to conventional therapies. This point is underscored by the observation that most of the interviewers, in case of unconventional therapies’ failure, come back to refer to Official Medicine - therefore demonstrating a substantial trust in the conventional practices. The gastrointestinal pathologies and upper respiratory tract are those ones for which most frequently parents recur to CAM. We have not to disregard their use to strengthen the immune system. These data agree with literature ones [10-14]. We cannot affirm the same in regards to the use of CAM for the chronic disease. The percentage of patients with chronic illness who use CAM is, in fact, very low compared to what we highlighted in other studies [21-28]. This result may be a bias due to the fact that few patients of our study were affected by chronic disease. The low number of patients with chronic diseases may be related to the fact that our survey has been performed by interviewing in-patient and out-patient parents of General Pediatric Hospitals, not in subspeciality departments. In fact patients with severe chronic illness – i.e neoplastic diseases, cystic fibrosis, hematological diseases, asthma – usually refer to specialist departments rather than to General Pediatric Hospitals.

Homeopathy and phytotherapy are the most common CAM used in children. In some reports the first one is preferred while in other surveys the second is prevalent. Other types of CAM - as acupuncture; massage therapy; osteopathy and chiropractic remedies - are less frequently preferred by parents [10-14,17,20-22]. In Calabria when parents choose CAM, the choice exclusively concentrates on phytotherapy or homeopathy, with a prevalence of the first. These data are disagree with those observed in Turin [37] and in the other Italian regions [7-9], in which homeopathy is more widely used. We must not forget that while in Calabria CAM are prescribed more frequently by pediatricians, Northern Italian parents often opt for “self-medication” of their children [37]. We may speculate that in our region pediatricians are more confident with phytoterapy than with homeopathy. Furthermore, we cannot exclude differences in local marketing forces.

The other forms of CAM were not reported in our research. This data is in contrast with our data obtained in Turin again, and with the other data coming from other countries showing that other modalities of CAM are used. We can therefore only speculate about the reason for this findings. It might be possible that the difference reflects the lack of professional figures in Calabria involved in other types for CAM as acupuncture or kyropractice.

The satisfaction’s level of the results which were achieved with CAM is high. These results agree with the previous data observed in Turin [37].

Remarkably we found that, in the most of cases, the pediatrician prescribes phytotherapic or homeopathic drug. This agrees with the data of the SIP’s research, which highlighted that over a quarter of the Italian pediatricians usually prescribe this kind of medicines [39].

As the use of CAM by children and adolescent is currently in a growth phase in all countries important pediatric scientific societies are interested in them. The American Academic of Pediatric includes Provisional Section on Complementary, Holistic and Integrative Medicine [40]. The Italian Society of Pediatrics established a working-party for CAM [39]. Italian pediatricians, cannot longer ignore this situation [41-43]. They need to improve their knowledge and communication skills about CAM in order to manage the parental attitude, with regard to the potential benefit and the potential interaction, in an open non-judgmental way. If this is not so, parents and children will turn to other professional and they risk to lose the central position in the children’s care.

## Competing interests

The authors declare that there is not conflict of interests

## Author details

Department of Medical Science and Surgery. Pediatric Unit.” Magna Graecia” University-Catanzaro.Italy.

## Authors’ contributions

TRD, FA, FZ,RM designed the study, provided data collection, wrote the manuscript. FZ designed the questionnaire and trained the interviewers. RM coordinated the work. All authors read and approved the final manuscript

## Other investigators

Dr. S. Braghò, Dr. M. Crinò, Dr. A. C. Oliverio, Dr.M.Filippo, Dr.D.Lucente.
